# Improving the Biological Value of Olive and Soybean Oil Blends with Olive Leaf Extract Obtained by Ultrasound-Assisted Extraction towards the Preparation of a Sauce Product

**DOI:** 10.3390/life11090974

**Published:** 2021-09-15

**Authors:** Mohammad Amin Aliyari, Karamatollah Rezaei

**Affiliations:** Department of Food Science, Engineering and Technology, Karaj Campus, College of Agriculture and Natural Resources, University of Tehran, Karaj 31587-77871, Iran; amin.aliyari@ut.ac.ir

**Keywords:** emulsion, oxidative stability, microbiological criteria, phenolic compounds, physicochemical characteristics

## Abstract

French sauce from different blends of soybean and olive oils was prepared and the oxidative stability of the optimum sauce sample, enriched with various amounts of olive leaf polyphenolic extract (OLE) (obtained via ultrasound-assisted extraction), was investigated over 90 days of storage. The microbiological and sensory properties of the samples containing the optimum amounts of OLE, as a substitution for synthetic preservatives, were studied. According to the results, the addition of olive oil at higher levels (75% and 100%) could affect the physicochemical properties of the sauce as compared to the control sample. It was also found that the addition of olive oil (up to 50%) would not significantly impact the sauce properties. Regarding the OLE enrichment in the samples, it was found that high levels of OLE could improve the oxidative stability of the samples. It was also found that OLE could be used as a preservative instead of commercial ones. Overall, this study suggests the potential use of olive oil and olive leaf extract in the preparation of French sauce to boost its nutritional value and its stability.

## 1. Introduction

Sauces and salad dressings are oil in water (O/W) emulsions that have drawn the attention of consumers for their daily diets. Sauces are used to improve the flavor, taste, and appearance of many food products and among them French sauce (FS) is one of the most well-known ones [1]. Generally, these types of products are called cold-sauces as any amount of heating treatment would result in an emulsion breakdown [2]. Increasing demand for health-promoting products has led salads to get special attention as healthy food products. This, in turn, has promoted the use of sauces as one of the main commodities in our daily diets. FS has been a matter of interest among scientists. For instance, Rasmussen et al. [3] showed the potential use of honey as a good substitute for EDTA and commercial sweeteners. In an another study, de Melo et al. [4] evaluated the stability and nutritional index of FS samples with the use of mannoprotein from spent brewer’s yeast. A typical FS usually consists of egg yolk, vinegar, oil, and tomato paste. In addition, several ingredients, such as paprika, mustard, and garlic powder, are needed as the main participants for sauces’ specific taste and flavor. Moreover, texturizing agents are normally used to form the consistency of the sauce [5]. In sauce formulations, the type and nature of the oil used are very important. In sauces and dressings, oil phase plays a vital role in their stability and general appearance. Additionally, the nutritional value of sauces can be considered as a direct function of their oil phase. Using oils with high amounts of saturated fatty acids can easily lead to several health problems such as obesity, cardiovascular disease, and cancer [6].

Olive oil is a source of beneficial components, such as polyphenolics, hydrocarbons, sterols, pigments, volatile compounds, and vitamins [7]. Olive oil is relatively resistant to oxidation because of its noticeable amount of monounsaturated fatty acids (MUFAs) as well as the presence of natural antioxidants such as α-tocopherol and phenolic compounds, such as tyrosol, hydroxytyrosol, and others [8]. Among the MUFAs, oleic acid contributes 58–83% of the fatty acids in olive oil, which has been proven to provide multiple beneficial impacts such as anti-inflammatory and anticancer effects [9]. Because of wide health benefits for olive oil, it can be used as a new oil phase in the preparation of O/W model emulsions such as FS and mayonnaise. In general, finding a new oil phase with functional and health properties which does not alter the physical and sensorial properties of the final product, has been a matter of interest among researchers for many years [10].

One of the most important factors in sauces and salad dressings is their stability. Because of their relatively high content of oil and perishable ingredients such as egg yolk, they are likely to spoil quickly [6]. Thus, in order to preserve and maintain them for a long time, the use of preservatives is usual in the sauce industry. The presence of commercial preservatives, such as sorbate and benzoate salts, is a major issue in these types of products; as they can cause several health problems such as allergic reactions or different cancers [11]. Using natural preservatives instead of synthetic ones is growing rapidly. For example, Rafiee et al. [12] used nanoliposomes which contained polyphenolic compounds of pistachio green hull as a natural preservative agent in a mayonnaise formulation. In an another study, Bruzewicz et al. [13] used baicalin (from baical skullcap root) to prepare mayonnaise samples to improve their shelf life.

Nowadays, food waste products are considered as excellent sources of bioactive compounds such as polyphenolics, protein residues, and fibers. Therefore, the possibility of using them in a wide variety of food formulations and also reducing the environmental burden is of high interest for both food technologists and environmental activists. Using food sector by-products provides major benefits to the larger world population by supplying additional food sources at lower costs [14,15,16]. Olive leaves, which are the by-products of olive processing industries, are considered to be a rich sources of polyphenolic compounds, which have been used for food, medicine, and cosmetic applications [17]. Oleuropein, which is classified as a phenolic bitter compound, is found abundantly in olive leaves and offers several health benefits, such as cardio-protection, neuro-protection, antiaging, anti-inflammatory, anticancer, antioxidant, and antimicrobial activities [18,19]. Tyrosol is another polyphenolic compound that is found in olive leaves at high quantities. Şahin and Bilgin [20] reported that the antioxidant activity of hydroxyl tyrosol is ten times as high as that of green tea polyphenolics. Because of the antimicrobial properties of olive leaves, they could be a promising natural ingredient to help enhance the shelf life of emulsion-based products. Additionally, due to the high polyphenolic content of olive leaves, they can also improve the overall nutritional value of products. Thus, it is crucial that the nutritional value of these products is assessed [21].

Physicochemical as well as oxidative and microbiological criteria are the most practical methods for analyzing emulsion-based products (mainly sauces) in terms of their nutritional value and stabilities. Considering the above points, the objectives of this study are to find the optimum amount of olive oil in combination with soybean oil (which is widely used in sauce industries) in the formulation of FS and to further apply olive leaf extract (OLE) obtained by ultrasound-assisted extraction (UAE) to improve the shelf life of FS. The possibility of using OLE instead of commercial synthetic preservatives (benzoate and sorbate salts) are also evaluated.

## 2. Materials and Methods

### 2.1. Materials

Egg yolk, sugar, salt, vinegar, citric acid, tomato paste, paprika, garlic, and mustard powders were purchased from a local supermarket in Tehran, Iran. Soybean oil, xanthan and guar gums were provided by Behrouz Nik Industrial Company (Karaj, Alborz Province, Iran). Virgin olive oil without any additive was purchased from Massoud Oil Company (city of Roudbar, Guilan province of Iran). Olive leaves were harvested from olive trees in Karaj, Iran in mid-June from the Koroneiki cultivar. All chemicals were of analytical grade and obtained from Merck Chemical Company (Darmstadt, Germany). Commercial FS was purchased from a local supermarket in Tehran, Iran and was from Bijan Industrial Company.

### 2.2. Chemical Analysis of Soybean and Olive Oils

Basic parameters of olive and soybean oils (acid value (AV), peroxide value (PV), iodine value (IV), and saponification value (SV)) were measured according to AOCS official methods [22]. The total phenolic contents (TPC) of the oils were measured according to Sousa et al. [23]. The chlorophyll content (ChC) and carotenoid content (CaC)) were determined using the method reported by Issaoui et al. [24]. Table 1 shows the basic parameters of oils used in this study.

### 2.3. Preparation and Characterization of Olive Leaf Extract

To obtain olive leaf extract (OLE), an ultrasound-assisted extraction technique (UAE) was applied according to the procedure outlined by Difonzo et al. [17]. At first, 25.0 g of olive leaves were placed in an oven at 120 °C until the moisture content of leaves reached below 1%. Then, the leaves were powdered in a mixer (IKA-WERKE M20, IKA Company, Staufen, Germany). After that, distilled water was added to the powdered leaves in the ratio of 1:20 (*w*/*v*, respectively). Then, the leaves and water blend were placed in an ultrasonic bath at 40 kHz (Universal Ultrasonic Cleaner, DSA 100-SK2, Fuzhou, China) and exposed to ultrasound for 90 min at 35 ± 5 °C. Then, the mixture was centrifuged (Hettich-Universal 320, Hettich Company, Tuttlingen, Germany) (25 °C, 30 min, and 4000× *g*). At the end, the supernatant was carefully separated and lyophilized. The obtained and lyophilized OLE was then stored at −20 °C for the rest of experiments.

The method reported by Şahin and Şamlı [25] was used with minor modifications to measure the total phenolic content (TPC) of OLE. The absorbance of the samples was measured at 765 nm. The results were expressed as mg Gallic acid equivalents (GAE) per gram of the dried olive leaves (mg GAE/g dried olive leaf). Gallic acid at different concentrations (0.170–0.150 mg/mL) was used to plot the calibration curve. A regression coefficient of 0.9983 was obtained for the calibration curve. The TPC of the obtained extract was determined as 27 ± 0.6 (mg GAE/g dry leaf).

### 2.4. Preparation of French Sauce Samples

The following proportions of the compounds (*w*/*w*) were used for the preparation of FS according to the method of Mizani et al. [1] with some modifications: egg yolk (20%), vinegar (10%), mustard powder (2.0%), garlic powder (0.16%), paprika powder (0.4%), tomato paste (4.0%), citric acid (0.1%), sugar (6.0%), salt (2.0%), xanthan gum (0.64%), guar gum (0.26%), sodium benzoate (0.063%), potassium sorbate (0.012%), water (16.4%), oil (38%). Olive and soybean oils were used at five different ratios (0%, 25%, 50%, 75%, and 100%, *w*/*w*). The sample containing 100% soybean oil and 0% olive oil was used as the control sample. All ingredients were mixed at a controlled temperature of 25 °C. For preparation of the samples, first, egg yolk was stirred to produce a considerable amount of foam. Then, vinegar, mustard powder, and citric acid were added and completely dissolved. Then, tomato paste and other ingredients except for gums were inserted and completely mixed. After that, xanthan and guar gums were added to make a proper texture. Finally, oil and water were added while mixing (from slow to fast) the components to generate a suitable emulsion structure in the samples. Samples were then maintained at 25 °C before the experiments.

### 2.5. Emulsion Stability

Emulsion stabilities of FS samples were measured using the method reported by Mun et al. [26] with slight modifications. Briefly, Falcon tubes containing 15 g of sauce samples were placed in a water bath (80 °C, 30 min). Then, the samples were cooled down for ~10 min, followed by centrifugation at 3000× *g* for 10 min. After centrifugation, the oil layer was carefully removed. Finally, the samples were weighed without the oil layer. The emulsion stability of the samples was determined using Equation (1):

(1) $$\mathrm{Emulsion}\;\mathrm{Stability}\;\left(\%\right)\;=\;\frac{F_{1}}{F_{0}}\times 100$$

where F_0_ and F_1_ are the sample weights before and after centrifugation, respectively.

### 2.6. Color Measurements

Color parameters (a*, b*, L*) were recorded at 25 °C using a Chroma Meter (CR-400, Konica Minolta Sensing, Tokyo, Japan). The Chroma Meter was placed at a distance of about 10 cm from each sample and the image was taken. After that, the data related to each color parameter appeared on the screen. All parameters were measured in triplicate. Images from different areas of each sample were taken.

### 2.7. Texture Profile Analysis

Using a texture analyzer TA-XT2 (Stable Micro Systems, Godalming, UK), firmness and consistency levels of the sauce samples were measured following the procedure described by Patil and Benjakul [27] at 25 °C. Briefly, samples were transferred to the containers up to the 125-mL level and a disc with a 35-mm diameter was used for compression. The speed was fixed at 1 mm/sec, until reaching a depth of 40 mm. To measure firmness and consistency, the force-time curve was used, in which the maximum point of the force curve and the area of the curve were used as the firmness and consistency values for the samples successively.

### 2.8. Rheological Properties

A rheometer (Physica MCR 301, Anton Paar, Graz, Austria) was used to determine the rheological properties of FS samples with parallel geometry (diameter: 25 mm, gap: 1 mm) using the probe PP 40/S (Anton Paar, Graz, Austria). First, the strain sweep from 0.01% to 100% at a constant frequency of 1.0 Hz was accomplished to determine the linear viscoelastic range. Then, the frequency sweep test in the 0.1–100 Hz frequency range was obtained under a constant strain of 0.5% and at 25 °C.

### 2.9. pH Determination

A 5% (*w*/*v*) sample solution was prepared and the pH values of the samples were measured using a pH meter (Crison-GLP 22, Crison Instruments, Alella, Spain) at 25 °C. All tests were carried out in triplicate.

### 2.10. Characterization of Enriched French Sauce Containing 50:50 (v/v) Olive and Soybean Oils and Different Amounts of Olive Leaf Extract

For further analysis, sauce samples were made using 50% olive oil and 50% soybean oil in the formulations (according to the obtained results, this blend of oils was selected as the optimum sample, so it was used for the rest of the experiments). To observe the effects of pure OLE on the oxidative parameters, commercial preservatives were not used in this stage. OLE was added to sauce samples at different levels (500, 1000, 1500, and 2000 mg/kg). A sample without the addition of OLE was used as the control. Sauce oxidative stability tests were then carried out every 15 days for a 3-month storage at 25 °C.

#### Oxidative Stability Evaluation

The AV, PV and *p*-anisidine values (PAV) were measured according to the official recommended methods of AOCS [22].

The total oxidation value (Totox) of each sample was determined using Equation (2):

Totox value = (2 × PV) + PAV(2)

All the oxidative stability tests were carried out under the daylight conditions.

### 2.11. Use of OLE in the Sauce Samples

In the last part of this study, the optimum amount of OLE was selected and the microbiological activities of a sauce sample with the optimum amount of OLE (FS-OLE) and without the addition of commercial preservatives, and a sample with commercial preservatives and without OLE, were measured. Sensory evaluations were also carried out on those samples as well as with a commercial product.

#### 2.11.1. Microbiological Control

Total plate counts for mesophilic aerobic bacteria, yeast and molds, and the presence of *E. coli* and lactic acid bacteria of two FS samples with the substitution of commercial preservatives/OLE, were determined in seven stages (at the beginning and every 15 days up to three months of storage). For each analysis, first, 10 g of FS sample was weighed in a test tube and homogenized with peptone water (90 mL). Then, suitable dilutions of each suspension (1.0 mL) were poured and spread on the total-count plates and selective agar plates (Merck, Darmstadt, Germany). The total plate counts of aerobic mesophilic bacteria were carried out by means of a standard plate count agar and incubated at 30 °C for 72 h. Yeast and molds were measured via YGC (yeast extract glucose chloramphenicol) agars, which were incubated at 25 °C for 6 days. In order to determine the presence of *E. coli*, 1 mL of each sample’s suspension was added to 9 mL of lauryl sulfate broth (LSB) and incubated at 37 °C for 48 h. Acid-tolerant microorganisms were identified using orange serum agar (OSA) at 45 °C, in which OSA was added to 1 mL of sample and after mixing it was left to solidify at room temperature, after which, it was incubated at 30 °C for 5 days.

#### 2.11.2. Sensory Analysis

Sensory analysis of three samples (with and without OLE and a commercial product) were performed according to the method explained by Liu, Xu, and Guo [28]. Briefly, a 9- point hedonic scale was used (1 = the poorest and 9 = the best). The characteristics of samples (appearance, odor, texture, color, taste, and overall acceptance) were evaluated by 25 panelists (21–30 years of age). Prior to the sensory evaluation, all panelists were familiar with FS as part of their own experiences. Bread and water were provided between testing the samples to reset the remaining taste from the previous samples.

### 2.12. Statistical Analysis

Statistical analyses among different experiments were carried out using the SPSS program for Windows (version 25.0, SPSS Inc., Chicago, IL, USA). The evaluation of the differences among the treatments were performed applying a one-way analysis of variance (ANOVA) with Duncan’s test at *p* < 0.05.

## 3. Results and Discussion

### 3.1. Emulsion Stability

Emulsion stability is an important factor in emulsion-based systems. This factor is related to some phenomena, such as coalescence, flocculation, and Ostwald ripening [26]. In sauces and salad dressings, proteins can make a layer around oil droplets. The generated layer by proteins can play a protective role against the accumulation of oil droplets in one area [29]. The results of the emulsion stability of samples are shown in Table 2. According to the results, all samples showed a very high emulsion stability (99.4–99.9%) at day 0 of production. As sauces and salad dressings contain high amounts of oil in their formulations, particles cannot move easily through their matrix, thus the stability of these products are usually high [30]. The stability levels of the samples were slightly decreased as the ratio of olive oil increased. The sample containing 100% olive oil (100OO) showed the lowest stability (99.4%). However, the control sample (100SO) showed the highest stability level (99.9%). From a statistical point of view, addition of olive oil up to 50% did not cause a meaningful difference from the control sample both on day 0 and on day 90 of the storage time. Olive oil generally contains less PUFAs than soybean oil. The partial reduction in the stability of the emulsion with an increase in olive oil is due to the oil’s natural fatty acid profile. Based on Magnusson et al. [31], a positive correlation was obtained between the amount of unsaturated fatty acids and the emulsion stability. There was a decreasing trend in the emulsion stabilities of the samples during the storage time, but the trend was much more obvious in the samples containing 75% and 100% olive oil (75OO and 100OO, respectively). This decreasing trend could be due to the sensitivity of saturated fatty acids to shear stress, so their crystalline structure can be damaged more easily [32].

### 3.2. pH Analysis

pH is one of the most important parameters in the evaluation of sauces and salad dressings. A high pH value (usually more than 4.2) usually causes serious damage such as growing pathogenic microorganisms (e.g., *Staphylococcus aureus*). Constant pH monitoring is one of the most critical factors in preparing new formulations of emulsion-based products [33]. The results of pH tests are shown in Table 3. Regarding the obtained results, the initial pH values (day 0) of the samples did not show significant differences. However, they decreased during the storage period of 90 days. Sample 100SO showed the lowest pH value (3.36), while the sample 75OO showed the highest pH of 3.97. Similar to the findings of the current study, Worrasinchai et al. [33] reported a declining trend of pH over 64 days of storage in mayonnaise samples. The reduction in pH during the storage time could be related to the breakdown of ester groups and generation of acidic groups [34]. Moreover, the growth and activity of some microorganisms, mainly lactic acid bacteria, can cause a drop in the pH [33].

### 3.3. Color Analysis

**Figure 1:**
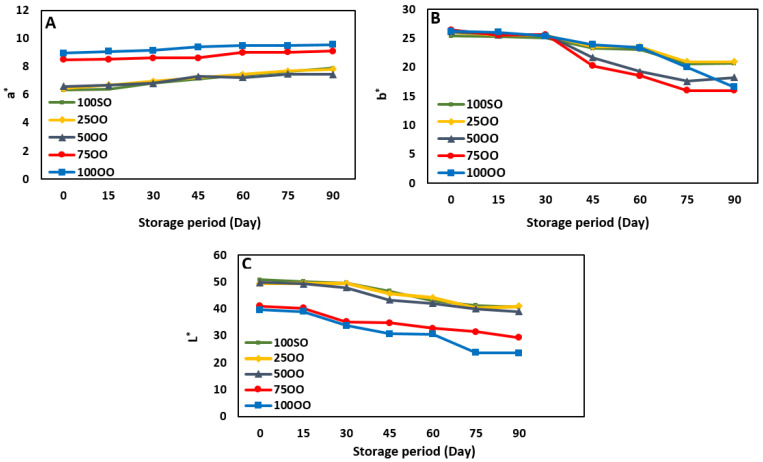
Color parameter ((**A**): a*: redness, (**B**): b*: yellowness, and (**C**): L*: lightness) changes in French sauce samples prepared with different blends of olive and soybean oil (100SO: samples with 100% soybean oil, 25OO: samples with 75% soybean oil and 25% olive oil, 50OO: samples with 50% soybean oil and 50% olive oil, 75OO: samples with 24% soybean oil and 75% olive oil, and 100OO: samples with 100% olive oil).

The collected data of color parameters including L* (lightness), b* (yellowness), and a* (redness) during the storage period are presented in Figure 1. On day 0, all samples showed different values for color parameters. Lightness is the most important color parameter in sauce products; as it directly affects consumer choices [26]. Results showed that the L* factor had the highest value in the control sample (100SO) indicating that it was the most transparent sample among the others. This value decreased as the relative ratio of olive oil increased, such that samples with 75% and 100% olive oil had major differences from the other samples. The differences in the L* value is due to the different light scattering of the components from different samples.

The scattering and absorption of samples are largely dependent on the reflective index, size and dispersion of droplets, concentration, and the presence of chromophobic materials [35]. As the soybean oil used in the current study was much paler than the olive oil (since soybean oil had a much lower content of pigments than did the olive oil, Table 1), the control sample showed maximum transparency (L* = 51). However, the sample 100OO showed the highest turbidity (L* = 40). Regarding the yellowness and redness factors, it can be clearly seen that the values increased with the increasing ratio of olive oil. The control sample showed the lowest values for b* and a* (25 and 6, respectively). In terms of b* value, there were no apparent differences among all samples on day 0 of production. The differences in the a* values, especially in the samples 75OO and 100OO, are due to the high amounts of intrinsic pigments in the olive oil [36]. On day 90 of storage, the lightness and yellowness values for all the samples were lowest, while the redness was somewhat higher. The rates of decrease in the samples containing higher levels of olive oil were higher if compared to that of the control sample. The polyphenolic content in olive oil is much higher than that in soybean oil (178 mg compared to 4 mg gallic acid equivalents/kg oil (Table 1)). As a result of the oxidation of polyphenolic compounds, the L* value can decrease faster in samples containing higher levels of olive oil. Additionally, the changes in the droplet sizes for the samples with different ratios of olive/soybean oils could be another reason for such changes. Generally, droplets get larger as the time proceeds towards the end of the storage period and therefore influences the light scattering of the samples [27]. This explanation was also confirmed by our emulsion stability results (Table 2), where a slight reduction was observed in the emulsion stability during the storage time. The b* values of all samples decreased on day 90 as a result of oxidation in the pigments [37]. Carotenoids (especially xanthophylls, which can be found in egg yolk and oils) are more sensitive to oxidation [38]. As a consequence, in samples containing higher levels of olive oil the degradation was much sharper than in the control. Badr [39] reported that the carotenoid content tends to decrease in liquid egg yolk during storage, but the rate of decrease at low temperatures is noticeably faster than at room temperature. The increase in the a* value during the storage time could be related to the oxidation of lipid components [27].

### 3.4. Textural Properties

The data obtained for the changes in the consistency and firmness of FS samples over the 90 days of storage are given in Table 4. On day 0, a decreasing trend was observed with an increase in the ratio of olive oil, which might be due to the lower amounts of unsaturated fatty acids in the samples containing higher levels of olive oil. Patil et al. [27] and Kupongsak et al. [40] reported similar results for the textural properties of mayonnaise at different ratios of fish/coconut and rice bran/coconut oil, respectively.

During the study period (90 days), both the firmness and consistency dropped for all FS samples (Table 4). This fact could be related to a phenomenon such as coalescence, where oil droplets tend to join together and build larger particles [41]. These findings agree well with the results of the emulsion stability for the FS (Table 2).

### 3.5. Rheological Properties

**Figure 2:**
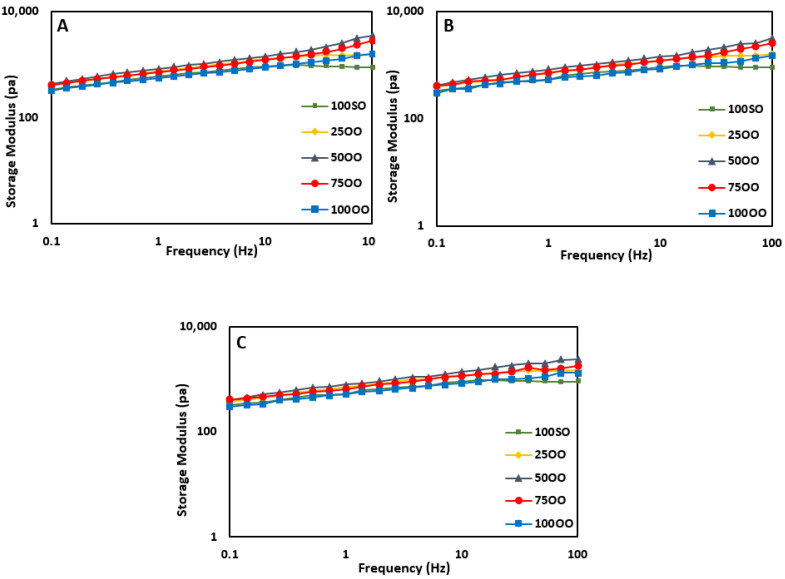
Storage modulus (G’) of French sauce samples prepared with different blends of olive/soybean oil ((**A**): Day 0, (**B**): Day 45, and (**C**): Day 90 of storage). 100SO: samples with 100% soybean oil, 25OO: samples with 75% soybean oil and 25% olive oil, 50OO: samples with 50% soybean oil and 50% olive oil, 75OO: samples with 25% soybean oil and 75% olive oil, and 100OO: samples with 100% olive oil.

**Figure 3:**
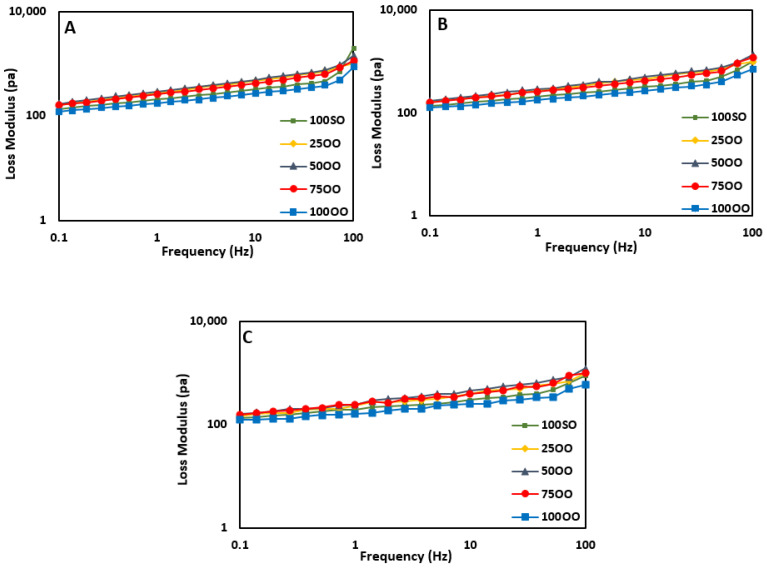
Loss modulus (G’’) of French sauce samples prepared with different blends of olive/soybean oil ((**A**): Day 0, (**B**): Day 45, and (**C**): day 90 of storage) (100SO: samples with 100% soybean oil, 25OO: samples with 75% soybean oil and 25% olive oil, 50OO: samples with 50% soybean oil and 50% olive oil, 75OO: samples with 25% soybean oil and 75% olive oil, and 100OO: samples with 100% olive oil).

The changes in the viscoelastic properties of FS samples are shown in Figure 2 (Storage modulus) and Figure 3 (Loss modulus). For all samples, the loss modulus (G’’) was lower than the storage modulus (G’). This indicates that all samples have an elastic behavior rather than a viscous behavior. Moreover, samples showed linear viscoelastic responses with increases in the angular frequency, indicating that all prepared FSs can be considered as a gel-like network [42].

On day 0, the storage moduli for all samples increased slightly with an increase in the angular frequency (Figure 2). The sample 50OO and the control (100SO) indicated the highest and lowest storage modulus, respectively. This shows that the sample with 50% olive oil had the best power of structural uniformity [38], meaning that it required higher shear stress to flow. This subject is a matter of importance in the pouring ability of sauces in factories as well as packages.

In terms of loss modulus (Figure 3), there were no clear differences among the samples. However, sample 50OO showed the highest value. Higher values of loss modulus indicate that the sample containing 50% olive oil had a better behavior of viscoelasticity compared to the others. On day 45 and day 90, all samples showed a slight decrease in the storage modulus compared to day 0. This means that the elastic behavior and the structural uniformity dropped in all samples and samples had more liquid-like behaviors compared to the freshly-prepared samples on day 0. These findings are in agreement with the emulsion stability data, where the stability was reduced as the bonds between the oil droplets started to weaken. No apparent changes were found in terms of loss modulus during the storage time (Figure 3a–c).

**Figure 4:**
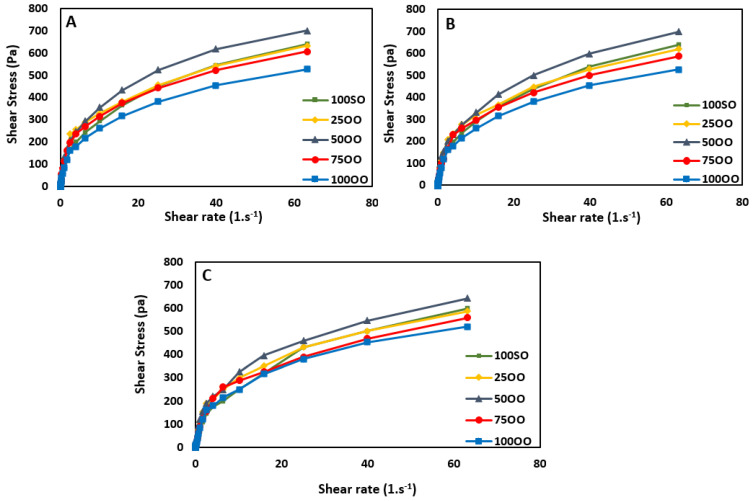
Flow curves of French sauce samples prepared with different blends of olive/soybean oil (**A**): Day 0, (**B**): Day 45, and (**C**): Day 90 of storage (100SO: samples with 100% soybean oil, 25OO: samples with 75% soybean oil and 25% olive oil, 50OO: samples with 50% soybean oil and 50% olive oil, 75OO: samples with 25% soybean oil and 75% olive oil, and 100OO: samples with 100% olive oil).

The flow curves of prepared FS samples with different ratios of olive/soybean oil are presented in Figure 4. The shear stress of all samples soared as the shear rate increased. However, there was no linearity in the relationship between the shear rate and shear stress for any of the sauce samples. Thus, all sauce samples can be considered as non-Newtonian liquids [43].

On day 0, sample 50OO showed the highest shear stress. This shows that it had the highest viscosity compared to the other samples; since the viscosity can be defined as the ratio of shear stress and shear rate. On days 45 and 90, all samples showed lower shear stress values compared to the freshly prepared samples on day 0. Generally, based on consumer data, higher values of shear stress as a function of shear rate are preferred [44]. Such products could cause a better mouth feel, but they could have some limitations considering the pumps and the fillers that are needed industrially [27].

### 3.6. Stability Evaluation

#### 3.6.1. Oxidative Parameters

The AVs of extracted lipid phases from the samples are shown in Table 5. Generally, AV measures the free fatty acids, which are made through the hydrolysis of lipids in the presence of water [45]. According to Table 5, the AV was increased in all samples during storage for 90 days, but the rates of increase were different among the different samples. The control sample (with no added OLE) showed the highest changes during storage, for which the AV was 1.1 at the beginning, but reached its maximum (4.9) at the end of the studied period. The increase in the AV during storage could be related to the activity of the hydrolytic enzymes in the eggs due to the acidic conditions of the sauce samples [46]. Additionally, the growth and activity of acid tolerant bacteria, especially lactic acid bacteria, could also impact on the free fatty acid formation in the aqueous phase of the samples [34]. The AV for the samples containing OLE, especially those with higher levels (1500 and 2000 mg/kg), increased at much lower rates than the control. Such effects can probably be mainly attributed to the polyphenolic compounds in the OLE, which were measured at 27 mg GAE/g dry leaf.

The results for changes in the PV during the storage period are shown in Table 6. The PV for all of the samples showed an initial increase followed by a slight decrease (except for the sample with 2000 mg/kg OLE) and then another increase afterward. The slight decrease, especially around day 30 and 45 of storage, is due to the breakdown of hydroperoxides that can form other compounds, and the high increase in the rate in samples around day 75 and 90 could be the result of boosting oxidative reactions [47]. Samples with higher amounts of OLE showed lower levels of PV than the control sample. While the sample containing 2000 mg/kg of OLE indicated the lowest PV value of 5.0 on day 90, the control sample showed the highest PV (8.9). These findings confirm the positive effect of OLE, especially its high and unique polyphenolic profile [48], on the sample’s shelf life. The obtained results are in agreement with those of other related studies in this area [49,50].

Generally, the oxidation process has a detrimental impact on products with high fat levels such as sauces. The reactions taking place in the interface of oil–water phases can speed up the process of oxidation [51].

PAV is another parameter that measures the secondary oxidative compounds, which are responsible for the undesirable flavors and odor in products. These oxidation reactions can lead to the formation of numerous compounds, especially 2-alkenals and 2,4-dienals [50]. Generally, high levels of PAV indicate the deterioration of lipids in the food matrix [52].

The PAV results of samples during the storage period are presented in Table 7. There was an increasing trend in the PAV during the storage time of 90 days. Such a trend could be related to the acceleration of oxidative processes with time. Samples containing higher levels of OLE (1500 and 2000 mg/kg) showed lower increases in the PAV. Timm-Heinrich et al. [53] reported that some polyphenolic compounds can minimize the production of oxidative products such as hexanal and heptadienal. On day 90 of storage in the current study, the control sample had the highest PAV, while the sample containing 2000 mg/kg of OLE showed the lowest PAV (12.6 and 5.7, respectively).

**Figure 5:**
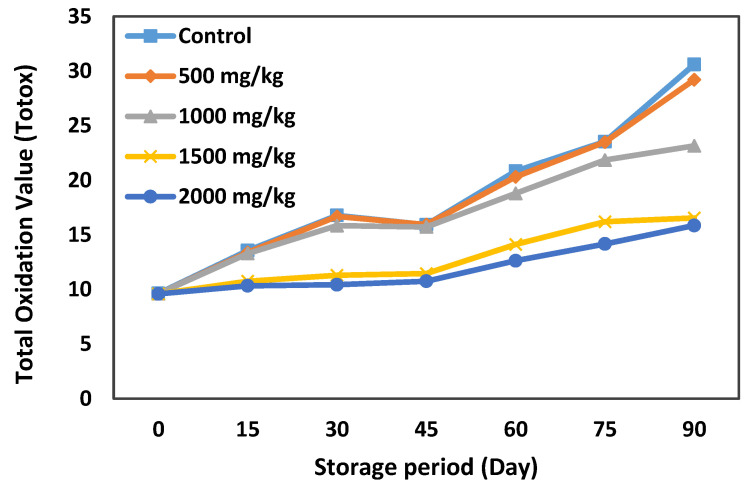
Changes in the totox value of French sauce samples enriched by different levels of olive leaf extract (OLE) (up to 2000 mg/kg) during 90 days of storage.

The total oxidation value (totox) is an index that is directly linked to both PV and PAV. Therefore, hydroperoxides and their primary and secondary oxidation products are reported by this parameter. The results of changes in the totox values of samples during the storage period are depicted in Figure 5. The totox values for all samples were increased by time. However, the samples with higher amounts of OLE (1500 and 2000 mg/kg) increased at lower rates (Figure 5). However, the control sample and sample with 500 mg/kg OLE experienced higher changes in terms of totox values. On day 90 of storage, the control sample indicated the highest totox value of 30.6 while the sample containing 2000 mg/kg OLE showed the lowest totox value of 15.8. Overall, the trend for changes in the totox value (Figure 5) is similar to that of PV and PAV (Table 6 and Table 7, respectively). The decreasing effect of OLE on totox values in the samples treated with high amounts of OLE can be related to the presence of polyphenolic groups in the extract that have inhibitory effects against the generation of primary and secondary oxidation products [50].

#### 3.6.2. Microbiological Properties

**Figure 6:**
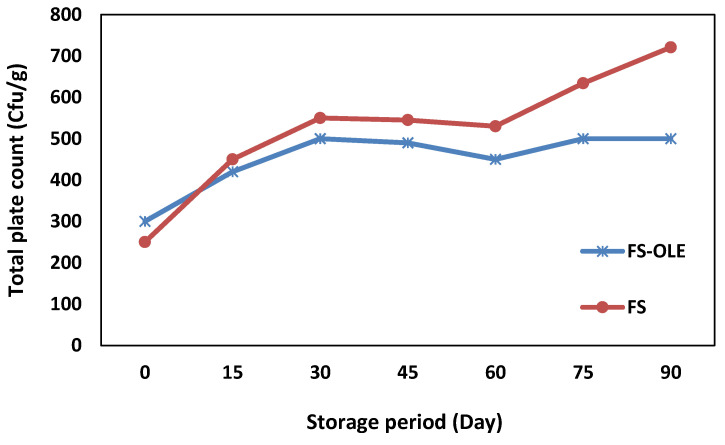
Total plate counts of mesophilic aerobic bacteria of sauce samples (FS-OLE: samples with 2000 mg/kg OLE and no commercial preservatives; FS: Samples with commercial preservatives, sodium benzoate, 0.063% (*w*/*w*), and potassium sorbate, 0.012% (*w*/*w*), without the addition of OLE).

The results of the total plate count for mesophilic aerobic bacteria of FS samples with and without using OLE are depicted in Figure 6. Both samples showed increases in the total plate counts during storage. However, the sample containing OLE at 2000 mg/kg indicated a lower value than the control. On day 0, the total plate count of the sample FS (i.e., that with no OLE added) was higher (300 cfu/g) than that in sample FS-OLE (containing OLE at 2000 mg/kg) (250 cfu/g). However, around day 15 of the storage, the total plate count for sample FS surpassed that of sample FS-OLE. Slight reductions were observed in the total counts of both samples around day 30 and 60 of storage, which could be due to the dissociation of acetic acid in the oil phase resulting in the depletion of microorganisms for a while [34]. The inhibitory effects of OLE on the growth of aerobic microorganisms can be due to its high levels of polyphenolic compounds that have been shown to have antibacterial and preservative effects in other studies [19,54]. Pourkomailian [55] reported that in samples with a high amount of oil phase, microorganisms usually stay in the oil phase and therefore they are not influenced by the pH effect of the aqueous phase.

A set of comparative microbiological data are shown in Table 8 for a sample containing 2000 mg/kg of OLE and no commercial preservatives, and a sample containing sodium benzoate at 0.063%, *w*/*w*, and potassium sorbate at 0.012%, *w*/*w*, as commercial synthetic preservatives, without the addition of OLE. According to these data, *E. coli* was not detected in either sample during the entire storage period. Moreover, the number of yeast and molds were similar in both samples (Table 8). Regarding acid tolerant bacteria, specifically lactic acid bacteria, they grew after day 60 of storage in sample FS; while in sample FS-OLE, they were not observed during the whole study period. These findings agree with the AV results of samples in Section 3.6 and confirms the protective role of olive leaves’ polyphenolic content against acid tolerant bacteria, which could have undesirable effects on the samples.

### 3.7. Sensory Evaluation

**Figure 7:**
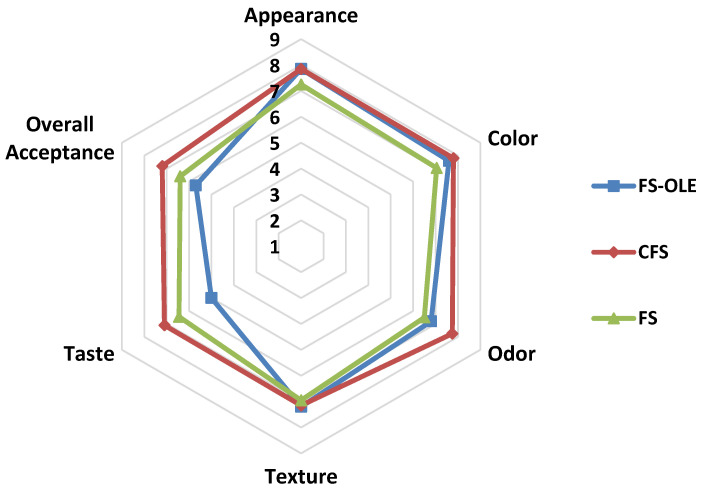
Sensory evaluation of French sauce samples (FS-OLE: samples with OLE, CFS: commercial French sauce, and FS: samples without OLE). Results are illustrated on a 9-point hedonic scale.

The results for the sensory evaluation of sauce samples with and without enrichment by OLE at 2000 mg/kg and a commercial FS are presented in Figure 7. No significant differences were found among the samples in terms of their appearances, colors, and textures. Apparently, this level of OLE does not significantly influence these sensorial attributes. Additionally, the use of olive oil at 50% did not change these parameters either. The sample without OLE showed the lowest score in odor, but there was no major difference between the samples. However, the commercial product gained the best score for odor. With regard to the taste scores, the commercial product obtained the best score, while the sample containing OLE had the lowest score, which was due to the intrinsic taste of OLE which could even be perceived at low concentrations. Regarding the overall acceptance scores, the commercial product received the highest average score of 7.2 but the sample without OLE resulted in a score of 6.4, which was significantly different from the sample containing OLE receiving a score of 5.7.

## 4. Conclusions

The major objective of this study was to produce a functional product replacing part of the soybean oil, which is currently used in the formulation of such products, with olive oil. Additionally, to improve the health aspects of the sauce, the enrichment of OLE in the sauce samples was investigated. Based on the results, there was a slight reduction in the emulsion stability of samples with increasing the amount of olive oil, especially at higher levels (75% and 100%). Moreover, the type and amount of the oils also affected the color parameters due to the different amounts of pigment available in the two studied oils. Textural and viscoelastic properties of samples were affected by the addition of olive oil. It was found that the sample prepared with 50% olive oil and 50% soybean oil was the best sample. Regarding the microbiological tests, it was understood that OLE was able to retard the growth of microorganisms during the storage time as well as prevent the growth of lactic acid bacteria at the end of the studied shelf-life time. So, OLE was found to be a promising alternative to commercial synthetic additives that are usually used in the formulation of sauces. Sensory analysis showed that OLE can influence the taste and odor even in small amounts, so further studies are needed to mask the intrinsic taste and odor of OLE in commercial food products.

## Figures and Tables

**Table 1:** Chemical analysis of soybean and olive oils (shown as SBO and OO, respectively) used in the experiments.

	AV	PV	SV	IV	TPC	ChC	CaC
**SBO**	0.1 ± 0.0	2.8 ± 0.6	195 ± 2	134 ± 1	4 ± 0.3	0.001 ± 0.000	0.025 ± 0.000
**OO**	1.5 ± 0.3	18.7 ± 0.2	191 ± 3	79 ± 2	178 ± 8	7.41 ± 0.00	2.16 ± 0.00

AV: acid value in mg NaOH/kg oil, PV: Peroxide value in meq O_2_/kg oil, SV: Saponification value in mg KOH/g oil, IV: Iodine value in g I_2_/100 g oil, TPC: Total phenolic content in mg GAE/kg oil, ChC: chlorophyll content in mg pheophytin, CaC: carotenoid content in mg lutein.

**Table 2:** Emulsion Stability (%) of Sauce samples prepared at different ratios of olive and soybean oils (100SO: samples with 100% soybean oil, 25OO: samples with 75% soybean oil and 25% olive oil, 50OO: samples with 50% soybean oil and 50% olive oil, 75OO: samples with 25% soybean oil and 75% olive oil, and 100OO: samples with 100% olive oil).

Storage Period (Day)	Sample				
	100SO	25OO	50OO	75OO	100OO
0	99.9 ± 0.1 ^Aa^	99.9 ± 0.1 ^Aa^	99.9 ± 0.1 ^Aa^	99.6 ± 0.1 ^Ab^	99.4 ± 0.0 ^Ac^
15	99.9 ± 0.0 ^Aa^	99.7 ± 0.2 ^ABa^	99.8 ± 0.1 ^Aa^	99.1 ± 0.1 ^Ab^	98.9 ± 0.1 ^Ab^
30	99.8 ± 0.0 ^Aa^	99.5 ± 0.1 ^Bab^	99.5 ± 0.1 ^ABab^	98.1 ± 0.8 ^Bb^	98.8 ± 0.1 ^Ac^
45	99.7 ± 0.1 ^ABa^	99.5 ± 0.0 ^Ba^	99.2 ± 0.2 ^Ba^	96.9 ± 0.1 ^Cb^	97.81 ± 0.7 ^Bc^
60	99.6 ± 0.2 ^Ba^	98.8 ± 0.0 ^Cb^	98.4 ± 0.1 ^Cb^	96.7 ± 0.7 ^Cc^	95.8 ± 0.1 ^Cd^
75	98.4 ± 0.1 ^Ca^	98.5 ± 0.1 ^CDb^	98.1 ± 0.1 ^Cc^	96.7 ± 0.1 ^Cd^	95.5 ± 0.5 ^Ce^
90	98.4 ± 0.3 ^Ca^	98.3 ± 0.3 ^Da^	98.1 ± 0.8 ^Ca^	96.6 ± 0.7 ^Cb^	95.4 ± 0.5 ^Cc^

The results are given as means ± SD, *n* = 3. a, b, c, d, e: In each row, values with the same letter (lower case) are not significantly different (*p* > 0.05). A, B, C, D: In each column, values with the same letter (upper case) are not significantly different (*p* > 0.05).

**Table 3:** pH values of French sauce samples prepared at different ratios of olive and soybean oils (100SO: samples with 100% soybean oil, 25OO: samples with 75% soybean oil and 25% olive oil, 50OO: samples with 50% soybean oil and 50% olive oil, 75OO: samples with 25% soybean oil and 75% olive oil, and 100OO: samples with 100% olive oil).

Storage Period (Day)	Sample				
	100SO	25OO	50OO	75OO	100OO
0	4.10 ± 0.10 ^Aa^	4.08 ± 0.08 ^Aa^	4.10 ± 0.05 ^Aa^	4.10 ± 0.10 ^Aa^	4.13 ± 0.02 ^Aa^
15	4.08 ± 0.08 ^Aa^	4.03 ± 0.05 ^Aa^	4.09 ± 0.01 ^Aa^	4.06 ± 0.05 ^ABa^	4.12 ± 0.00 ^Aa^
30	4.05 ± 0.05 ^ABa^	4.04 ± 0.05 ^Aa^	4.09 ± 0.00 ^Aa^	4.06 ± 0.05 ^ABa^	4.11 ± 0.00 ^Aa^
45	3.89 ± 0.09 ^Bb^	3.70 ± 0.00 ^Ba^	3.90 ± 0.10 ^ABb^	4.03 ± 0.05 ^ABc^	4.11 ± 0.01 ^Ac^
60	3.60 ± 0.10 ^Cab^	3.50 ± 0.10 ^Ca^	3.83 ± 0.28 ^BCbc^	4.01 ± 0.00 ^Ac^	4.00 ± 0.00 ^Bc^
75	3.50 ± 0.10 ^CDa^	3.46 ± 0.05 ^Ca^	3.63 ± 0.15 ^Ca^	3.97 ± 0.06 ^Bb^	3.90 ± 0.10 ^Cb^
90	3.36 ± 0.06 ^Da^	3.45 ± 0.05 ^Ca^	3.86 ± 0.05 ^ABCb^	3.97 ± 0.06 ^Bb^	3.90 ± 0.10 ^Cb^

The results are given as means ± SD, *n* = 3. a, b, c: In each row, values with the same letter (lower case) are not significantly different (*p* > 0.05). A, B, C, D: In each column, values with the same letter (upper case) are not significantly different (*p* > 0.05).

**Table 4:** Textural properties of French sauce samples prepared with different blends of olive and soybean oil (100SO: samples with 100% soybean oil, 25OO: samples with 75% soybean oil and 25% olive oil, 50OO: samples with 50% soybean oil and 50% olive oil, 75OO: samples with 25% soybean oil and 75% olive oil, and 100OO: samples with 100% olive oil).

Sample	Firmness (g)			Consistency (g.s)		
	Day 0	Day 45	Day 90	Day 0	Day 45	Day 90
100SO	163 ± 9 ^Aa^	154 ± 3 ^Aa^	128 ± 3 ^ABb^	2507 ± 22 ^Aa^	2303 ± 16 ^Ab^	1938 ± 16 ^Ac^
25OO	154 ± 5 ^ABa^	146 ± 7 ^Aa^	127 ± 3 ^ABb^	2474 ± 15 ^Aa^	2284 ± 18 ^Ab^	1921 ± 22 ^Ac^
50OO	161 ± 5 ^Aa^	152 ± 2 ^Aa^	131 ± 8 ^Ab^	2407 ± 12 ^Ba^	2180 ± 21 ^Bb^	1952 ± 28 ^Ac^
75OO	145 ± 4 ^BCa^	130 ± 2 ^Bb^	121 ± 9 ^ABb^	2034 ± 27 ^Ca^	1952 ± 17 ^Cb^	1509 ± 11 ^Bc^
100OO	134 ± 7 ^Ca^	121 ± 4 ^Cb^	116 ± 3 ^Bb^	1829 ± 14 ^Da^	1522 ± 11 ^Db^	1488 ± 17 ^Bc^

The results are given as means ± SD, *n* = 3. a, b, c: In each row, values with the same letter (lower case) are not significantly different (*p* > 0.05). A, B, C, D: In each column, values with the same letter (upper case) are not significantly different (*p* > 0.05).

**Table 5:** Acid values (mg NaOH/g) obtained for the samples of French sauce (50OO) enriched by different amounts of olive leaf extract (OLE).

Storage Period (Day)	OLE Concentration (mg/kg)				
	0	500	1000	1500	2000
0	1.1 ± 0.0 ^Aa^	1.1 ± 0.0 ^Aa^	1.1 ± 0.0 ^Aa^	1.1 ± 0.0 ^Aa^	1.1 ± 0.0 ^Aa^
15	1.2 ± 0.0 ^Bab^	1.2 ± 0.0 ^Ab^	1.2 ± 0.01 ^Aab^	1.1 ± 0.1 ^Aa^	1.1 ± 0.0 ^Aa^
30	1.4 ± 0.0 ^Ca^	1.4 ± 0.0 ^Ba^	1.3 ± 0.0 ^Aa^	1.3 ± 0.0 ^Ba^	1.3 ± 0.0 ^Ba^
45	2.3 ± 0.0 ^Db^	2.3 ± 0.1 ^Cab^	2.3 ± 0.0 ^Bab^	2.3 ± 0.0 ^Cb^	2.2 ± 0.0 ^Ca^
60	3.1 ± 0.0 ^Ec^	3.1 ± 0.1 ^Dc^	3.1 ± 0.0 ^Cc^	2.9 ± 0.0 ^Db^	2.6 ± 0.1 ^Da^
75	3.7 ± 0.1 ^Fb^	3.6 ± 0.1 ^Eb^	3.6 ± 0.4 ^Db^	3.0 ± 0.0 ^Da^	2.9 ± 0.0 ^Ea^
90	4.9 ± 0.0 ^Gd^	5.0 ± 0.0 ^Fd^	4.7 ± 0.1 ^Ec^	3.7 ± 0.2 ^Eb^	3.1 ± 0.1 ^Fa^

The results are given as means ± SD, *n* = 3. a, b, c, d: In each row, values with the same letter (lower case) are not significantly different (*p* > 0.05). A, B, C, D, E, F, G: In each column, values with the same letter (upper case) are not significantly different (*p* > 0.05).

**Table 6:** Peroxide values (meq O_2_/kg) of French sauce (50OO) samples enriched by olive leaf extract (OLE).

Storage Period (Day)	OLE Concentration (mg/kg)				
	0	500	1000	1500	2000
0	3.1 ± 0.0 ^Aa^	3.1 ± 0.0 ^Aa^	3.1 ± 0.0 ^Aa^	3.1 ± 0.0 ^Aa^	3.1 ± 0.0 ^Aa^
15	3.8 ± 0.0 ^Ba^	3.8 ± 0.0 ^Ba^	3.7 ± 0.0 ^BCb^	3.4 ± 0.0 ^Bc^	3.2 ± 0.1 ^Bd^
30	4.4 ± 0.0 ^Ca^	4.3 ± 0.1 ^Ca^	3.9 ± 0.0 ^Cb^	3.6 ± 0.1 ^Dc^	3.3 ± 0.0 ^Bd^
45	3.9 ± 0.1 ^Bab^	3.9 ± 0.1 ^Ba^	3.8 ± 0.0 ^Bb^	3.5 ± 0.1 ^Cc^	3.3 ± 0.0 ^Bd^
60	4.9 ± 0.1 ^Da^	4.7 ± 0.1 ^Db^	4.5 ± 0.1 ^Dc^	4.2 ± 0.1 ^Ed^	3.8 ± 0.0 ^Ce^
75	6.1 ± 0.1 ^Ea^	6.1 ± 0.0 ^Ea^	5.9 ± 0.1 ^Eb^	5.1 ± 0.1 ^Fc^	4.5 ± 0.1 ^Dd^
90	8.9 ± 0.0 ^Fa^	8.3 ± 0.2 ^Fb^	6.1 ± 0.1 ^Fc^	4.9 ± 0.0 ^Gd^	5.0 ± 0.1 ^Ed^

The results are given as means ± SD, *n* = 3. a, b, c, d, e: In each row, values with the same letter (lower case) are not significantly different (*p* > 0.05). A, B, C, D, E, F, G: In each column, values with the same letter (upper case) are not significantly different (*p* > 0.05).

**Table 7:** *p*-Anisidine values (mmol/kg) of French sauce samples (50OO) enriched by different amounts of olive leaf extract (OLE).

Storage Period (Day)	OLE Concentration (mg/kg)				
	0	500	1000	1500	2000
0	3.3 ± 0.0 ^Aa^	3.3 ± 0.01 ^Aa^	3.3 ± 0.0 ^Aa^	3.3 ± 0.0 ^Aa^	3.3 ± 0.0 ^Aa^
15	5.9 ± 0.1 ^Bc^	5.8 ± 0.0 ^Bc^	5.84 ± 0.1 ^Bc^	4.0 ± 0.1 ^ABb^	3.8 ± 0.1 ^Ba^
30	8.0 ± 0.0 ^Cb^	7.9 ± 0.1 ^Cb^	7.9 ± 0.8 ^Cb^	3.9 ± 0.9 ^ABa^	3.84 ± 0.1 ^Ba^
45	8.1 ± 0.0 ^Cb^	8.0 ± 0.0 ^Cb^	8.0 ± 0.0 ^Cb^	4.4 ± 0.1 ^Ba^	4.2 ± 0.3 ^Ca^
60	10.9 ± 0.0 ^Dd^	10.8 ± 0.0 ^Dd^	9.8 ± 0.6 ^Db^	5.6 ± 0.6 ^Ca^	5.0 ± 0.0 ^Da^
75	11.3 ± 0.0 ^Ed^	11.2 ± 0.0 ^Ed^	10.0 ± 0.0 ^Dc^	6.0 ± 0.0 ^CDb^	5.1 ± 0.1 ^Da^
90	12.6 ± 0.0 ^Fd^	12.6 ± 0.0 ^Fd^	10.9 ± 0.5 ^Ec^	6.6 ± 0.7 ^Db^	5.7 ± 0.2 ^Ea^

The results are given as means ± SD, *n* = 3. a, b, c, d: In each row, values with the same letter (lower case) are not significantly different (*p* > 0.05). A, B, C, D, E, F: In each column, values with the same letter (upper case) are not significantly different (*p* > 0.05).

**Table 8:** Total plate counts (cfu/g) of sauce samples during 90 days of storage.

	FS-OLE			FS		
	Yeast and Molds	*E. coli*	Lactic Acid Bacteria	Yeast and Molds	*E. coli*	Lactic Acid Bacteria
**0**	<100	ND	ND	<100	ND	ND
**15**	<100	ND	ND	<100	ND	ND
**30**	<100	ND	ND	<100	ND	ND
**45**	<100	ND	ND	<100	ND	ND
**60**	<100	ND	ND	<100	ND	4.0 × 10^2^
**75**	<100	ND	ND	<100	ND	4.2 × 10^2^
**90**	<100	ND	ND	<100	ND	4.2 × 10^2^
